# Effect of Increased Level of Lipoprotein(a) on Cardiovascular Outcomes in Patients With Ischemic Heart Disease: A Systematic Review and Meta-Analysis

**DOI:** 10.7759/cureus.72776

**Published:** 2024-10-31

**Authors:** Insha H Hamid, Neeharika Muppa, Dhruvi Modi, Sindhuja Sompalli, Ihtisham Habib, Sandipkumar S Chaudhari, Muhammad Arsalan, Danish Allahwala

**Affiliations:** 1 Physiology, Ghulam Muhammad Mahar Medical College (GMC), Srinagar, IND; 2 Medicine, St. George’s University, School of Medicine, St. George’s, GRD; 3 Internal Medicine, Gujarat Adani Institute of Medical Sciences, Bhuj, IND; 4 Internal Medicine, Jagadguru Sri Shivarathreeshwara (JSS) Medical College, Hyderabad, IND; 5 Internal Medicine, Medical Teaching Institute, Lady Reading Hospital Peshawar, Peshawar, PAK; 6 Cardiothoracic Surgery, University of Alabama at Birmingham, Birmingham, USA; 7 Family Medicine, University of North Dakota School of Medicine and Health Sciences, Fargo, USA; 8 Internal Medicine, Medical Teaching Institute, Lady Reading Hospital, Peshawar, PAK; 9 Nephrology, Fatima Memorial Hospital, Karachi, PAK

**Keywords:** cardiovascular, ischemic heart disease, lipoprotein a, mortality, systematic review and meta-analysis

## Abstract

Lipoprotein(a) (Lp(a)) has emerged as a significant cardiovascular risk factor, particularly in patients with ischemic heart disease (IHD). This systematic review and meta-analysis aimed to synthesize evidence on the impact of Lp(a) levels on cardiovascular outcomes in IHD patients. A comprehensive literature search was conducted across multiple databases, covering publications from January 2016 to October 2024. Studies assessing the relationship between Lp(a) levels and cardiovascular outcomes in IHD patients were included. The primary outcomes were major adverse cardiovascular events (MACE), all-cause mortality, myocardial infarction, and revascularization. Quality assessment was performed using the Newcastle-Ottawa Scale. Fourteen studies (five prospective, nine retrospective) met the inclusion criteria, with sample sizes ranging from 350 to 18,544 participants. Pooled analysis revealed that elevated Lp(a) levels were significantly associated with increased risk of MACE (HR: 1.31, 95% CI: 1.19-1.45), all-cause mortality (HR: 1.23, 95% CI: 1.15-1.31), myocardial infarction (HR: 1.20, 95% CI: 1.06-1.35), and revascularization (HR: 1.23, 95% CI: 1.08-1.39) in IHD patients. Sensitivity analyses confirmed the robustness of these findings. This meta-analysis provides strong evidence that elevated Lp(a) levels are associated with adverse cardiovascular outcomes in IHD patients. The findings underscore the potential role of Lp(a) as an important prognostic marker and suggest that incorporating Lp(a) assessment into clinical practice could enhance risk stratification. Future research should focus on establishing optimal Lp(a) cutoff values and evaluating the impact of Lp(a)-lowering therapies on cardiovascular outcomes in this high-risk population.

## Introduction and background

Lipoprotein(a) (Lp(a)) is an emerging cardiovascular risk factor that has garnered significant attention in recent years due to its strong associations with various cardiovascular diseases, including ischemic heart disease (IHD) [1]. Elevated Lp(a) levels have been identified as an independent risk factor for atherosclerotic cardiovascular disease (ASCVD), potentially contributing to adverse outcomes in patients with IHD. Ischemic heart disease, characterized by reduced blood flow to the heart muscle due to narrowed coronary arteries, remains a leading cause of morbidity and mortality worldwide [2-3]. Despite advances in preventive and therapeutic strategies, the residual risk of cardiovascular events persists in many patients with IHD, which necessitates further exploration of non-traditional risk factors like Lp(a) [4].

Lp(a) is a complex lipoprotein particle, consisting of an apolipoprotein B (ApoB)-containing low-density lipoprotein (LDL) molecule attached to a unique glycoprotein, apolipoprotein(a) (Apo(a)) [5-6]. Genetic factors predominantly determine Lp(a) levels, and they vary widely across individuals and ethnic groups. Unlike other lipoproteins, Lp(a) levels are minimally affected by lifestyle factors such as diet and physical activity, making it a challenging target for intervention [7]. Epidemiological studies have consistently shown that elevated Lp(a) levels are associated with an increased risk of cardiovascular events, including myocardial infarction, stroke, and peripheral arterial disease, suggesting its potential role as a causal factor in atherosclerosis and thrombosis [8].

The clinical relevance of Lp(a) has spurred interest in its measurement and potential as a therapeutic target. Current guidelines from organizations like the American Heart Association (AHA) and the European Society of Cardiology (ESC) recommend Lp(a) testing for individuals with a family history of premature ASCVD or elevated risk profiles that are unexplained by traditional factors [9]. However, there is still no consensus on specific Lp(a) cutoff values that would necessitate intervention in IHD patients [10]. Several therapeutic agents, including PCSK9 inhibitors, niacin, and, more recently, antisense oligonucleotides, have been explored for their ability to lower Lp(a) levels. Early-phase clinical trials have demonstrated promising results with these therapies, particularly with the novel agents that target Lp(a) synthesis specifically [11].

The objective of this systematic review and meta-analysis is to synthesize existing evidence on the effect of Lp(a) levels on cardiovascular outcomes in patients with ischemic heart disease. By examining the associations between elevated Lp(a) and adverse cardiovascular events, this review aims to elucidate the potential role of Lp(a) as a prognostic marker and therapeutic target in IHD. Understanding the impact of Lp(a) on cardiovascular outcomes could have significant implications for risk stratification and the development of targeted therapies to improve outcomes in this high-risk population.

## Review

Methodology

Literature Search

A comprehensive and systematic literature search was conducted across multiple electronic databases, including PubMed, Embase, and the Cochrane Library, covering publications from January 1, 2016 to October 25, 2024. Additional searches were carried out in Scopus and Web of Science to capture relevant studies that were not indexed in the primary databases. The search strategy employed a combination of controlled vocabulary, such as MeSH terms, and relevant keywords, including terms related to lipoprotein(a), cardiovascular outcomes, ischemic heart disease, and synonyms. The search strings were tailored for each database to ensure precision, incorporating both specific terms like “lipoprotein(a)” and broader terms such as “coronary artery disease” and “cardiovascular events.” To identify additional studies, reference lists of included articles and relevant review articles were manually screened. Grey literature, such as conference abstracts, theses, and clinical trial registries, was also examined to minimize publication bias. No restrictions were placed on language. Search results were imported into a reference management software, and duplicates were removed before proceeding with the study selection.

Study Selection

The study selection process was undertaken in two stages: title/abstract screening and full-text screening. Two independent reviewers screened the titles and abstracts of all identified studies based on predefined inclusion and exclusion criteria. Studies that met the inclusion criteria or where relevance could not be determined from the abstract alone were retained for full-text review. In the second stage, the same reviewers independently assessed the full texts to confirm eligibility, ensuring that studies included patients with ischemic heart disease and reported cardiovascular outcomes in relation to lipoprotein(a) levels. Studies were included if they were assessing the impact of Lp(a) on IHD and assessed one of the following outcomes: major adverse cardiovascular events (MACE), all-cause mortality, myocardial infarction, and revascularization. Articles were excluded if they included subjects other than IHD. We also excluded case reports, case series, reviews, and editorials. We also excluded studies where hazard ratio (HR) was not reported. Discrepancies between reviewers were resolved through discussion and, when necessary, consultation with a third reviewer. The study selection process was documented using a Preferred Reporting of Systematic Review and Meta-Analysis (PRISMA) flow diagram, providing a transparent account of the number of records identified, screened, assessed for eligibility, and included in the final analysis.

Data Extraction 

Data were extracted from each included study by two independent reviewers using a standardized data extraction form. Extracted data included study characteristics (such as author, year, country, and study design), sample size, and outcomes. Where available, additional data on potential confounders and adjustments made in statistical analyses were also recorded. To ensure accuracy and consistency, the reviewers compared extracted data, and any discrepancies were resolved through consensus or by consulting a third reviewer. Authors of the original studies were contacted when clarification or additional information was required.

Quality Assessment Using the Newcastle-Ottawa Scale

The methodological quality of the included observational studies was assessed using the Newcastle-Ottawa Scale (NOS), which evaluates studies across three domains: selection, comparability, and exposure (for cohort studies) or outcome (for case-control studies). Two reviewers independently assessed each study, scoring them based on criteria such as representativeness of the exposed cohort, ascertainment of exposure, comparability of study groups, and adequacy of follow-up. Each study was awarded a maximum of nine stars based on the NOS criteria, with higher scores indicating better methodological quality. Any disagreements between reviewers on quality assessment were resolved through discussion, with a third reviewer available for arbitration if necessary. The results of the quality assessments were summarized and used to interpret the strength and validity of the meta-analysis findings.

Data Analysis 

RevMan Version 4.5.1 was employed to calculate the pooled effect of Lp(a) on the outcomes assessed in this meta-analysis. Hazard ratios (HRs) were determined along with 95% confidence intervals (CIs). Adjusted HRs were utilized, and in cases where they were unavailable, unadjusted HRs were used. To investigate potential sources of high heterogeneity, we conducted a sensitivity analysis. Heterogeneity was quantified using the I-square (I²) statistic; if the I² value was 50% or higher, a random-effects model was applied to calculate pooled estimates. For lower heterogeneity, a fixed-effects model was used. The threshold for statistical significance was set at a p-value of 0.05.

Results

**Figure 1:**
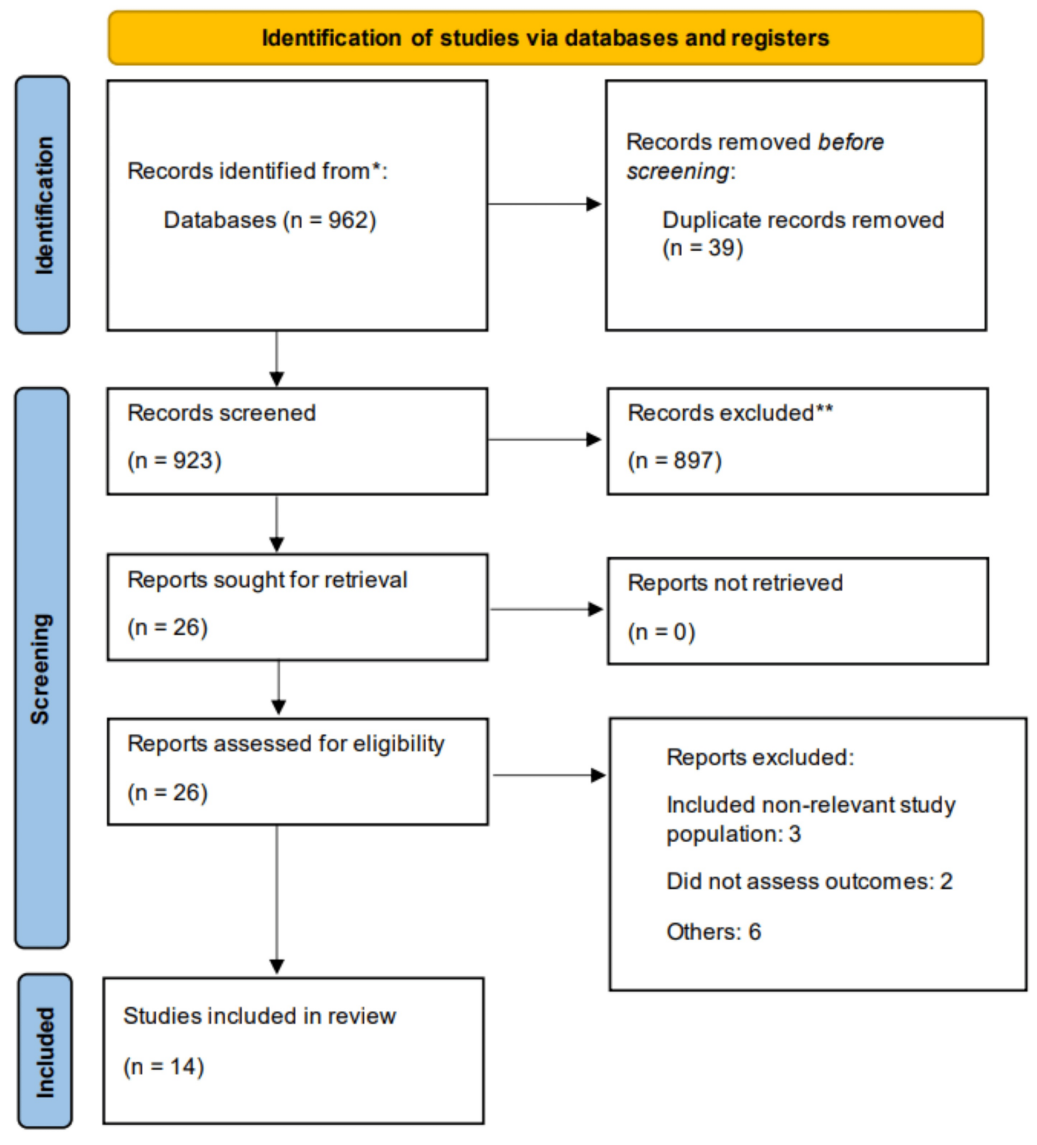
PRISMA flowchart of study selection PRISMA: Preferred Reporting of Systematic Review and Meta-Analysis. *Using predefined search criteria. **Using predefined inclusion and exclusion criteria.

Figure 1 presents the study selection process. A total of 962 studies were initially identified through the literature search. After removing 39 duplicates, the remaining studies underwent an initial screening based on titles and abstracts, from which 26 studies were selected for full-text assessment. Following a thorough evaluation, 14 studies met the inclusion criteria and were included in the meta-analysis. Of these, five studies were prospective in design, while the remaining nine were retrospective. The characteristics of the included studies varied in terms of geographical distribution, sample size, and study design. Notably, the studies represented a diverse set of countries, with multiple studies conducted in China and Japan. Table 1 presents the characteristics of included studies. The included studies were published between 2016 and 2024. The individual sample sizes ranged from 350 to 18,544 participants, reflecting a wide variation across studies. Table 2 presents quality assessment of included studies.

**Table 1:** Included studies characteristics

Author	Year	Design	Region	Sample Size	Follow-up
Amin et al. [12]	2024	Prospective	India	600	18 months
Cui et al. [13]	2022	Prospective	China	10,059	28.8 months
Dykun et al. [14]	2022	Retrospective	Germany	4,941	37.2 months
Hishikari et al. [15]	2020	Retrospective	Japan	410	24 months
Konishi et al. [16]	2016	Retrospective	Japan	904	56.4 months
Liu et al. [17]	2020	Prospective	China	4,078	12 months
Liu et al. [18]	2020	Retrospective	China	350	58.8 months
Suwa et al. [19]	2017	Retrospective	Japan	1,336	64 months
Xu et al. [20]	2018	Prospective	China	427	24 months
Yang et al. [21]	2022	Retrospective	China	765	17 months
Yoon et al. [22]	2021	Retrospective	Korea	12,064	88.8 months
Yuan et al. [23]	2024	Prospective	China	18,544	38.4 months
Zhang et al. [24]	2024	Retrospective	Italy	1,168	36 months
Zhu et al. [25]	2021	Retrospective	China	6,601	24 months

**Table 2:** Quality assessment of included studies

Author	Selection	Comparability	Ascertainment	Overall
Amin et al. [12]	2	3	3	Good
Cui et al. [13]	3	2	3	Good
Dykun et al. [14]	2	1	2	Fair
Hishikari et al. [15]	3	2	3	Good
Konishi et al. [16]	3	2	2	Good
Liu et al. [17]	3	2	2	Good
Liu et al. [18]	4	2	3	Good
Suwa et al. [19]	3	2	3	Good
Xu et al. [20]	3	1	2	Fair
Yang et al. [21]	4	2	2	Good
Yoon et al. [22]	3	3	2	Good
Yuan et al. [23]	4	2	3	Good
Zhang et al. [24]	4	2	3	Good
Zhu et al. [25]	3	2	2	Good

Major adverse cardiovascular events

**Figure 2:**
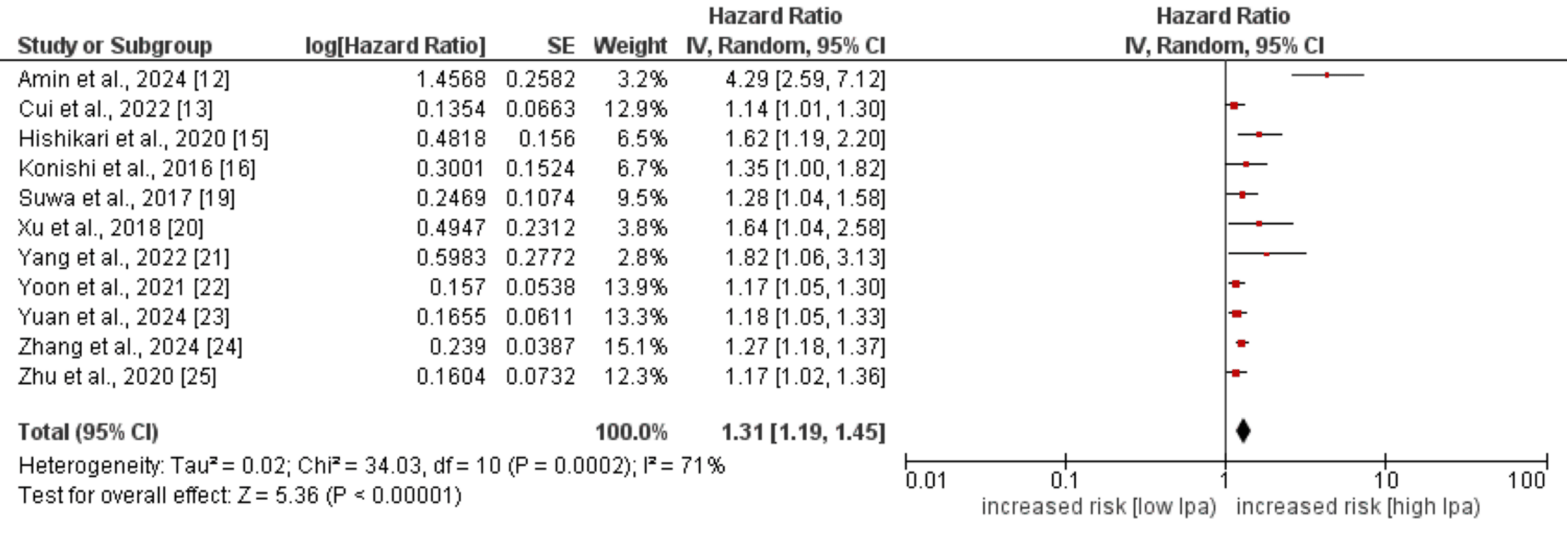
Effect of Lp(a) on MACE Lp(a): lipoprotein(a), MACE: major adverse cardiovascular events. References [12-13,15-16,19-25].

Eleven out of 14 studies assessed the effect of Lp(a) on MACE in patients with IHD. Pooled results are depicted in Figure 2. As shown by pooled analysis, the high Lp(a) is associated with an increased risk of MACE events among patients with high Lp(a) (HR: 1.31, 95% CI: 1.19-1.45, p-value=0.002). High heterogeneity was reported among the study results (I^2^: 71%). We performed sensitivity analysis to find the source of high heterogeneity despite the fact that all studies showed similar association between Lp(a) and MACE. By removing the study conducted by Amin et al. [12], the pooled effect remains similar but the heterogeneity decreased from 71% to 16% (HR: 1.23, 95% CI: 1.17-1.30, p-value= 0.001).

All-cause Mortality

**Figure 3:**
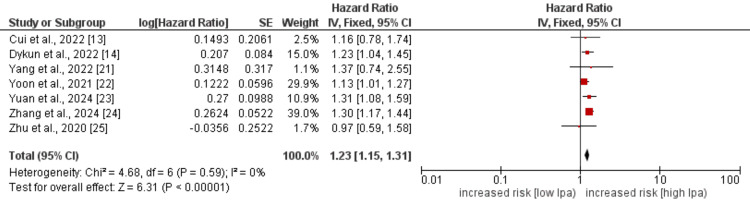
Effect of Lp(a) on all-cause death Lp(a): lipoprotein(a). References [13-14,21-25].

Seven studies were used in the pooled analysis to determine the effect of high Lp(a) levels of all-cause mortality in IHD patients, and the pooled analysis findings are presented in Figure 3. Pooled analysis showed that the hazard of all-cause mortality was significantly higher in patients with high levels of Lp(a) (HR: 1.23, 95% CI: 1.15-1.31, p-value<0.001). Low heterogeneity was reported among the study results that showed the consistent effect of high levels of Lp(a) across all studies.

Myocardial Infarction 

**Figure 4:**
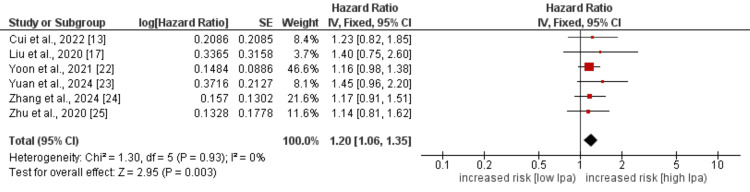
Effect of Lp(a) on myocardial infarction Lp(a): lipoprotein(a). References [13,17,22-25].

Six studies were used in the pooled analysis to determine effect of high Lp(a) levels on myocardial infarction in IHD patients, and the pooled analysis findings are presented in Figure 4. Pooled analysis showed that the hazard of myocardial infarction was significantly higher in patients with high levels of Lp(a) (HR: 1.20, 95% CI: 1.06-1.35, p-value: 0.003). Low heterogeneity was reported among the study results that showed the consistent effect of high levels of Lp(a) across all studies.

Revascularization

**Figure 5:**
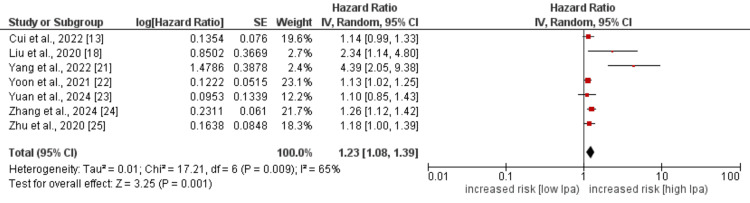
Effect of Lp(a) on revascularization Lp(a): lipoprotein(a). References [12,18,21-25].

Seven studies assessed the effect of high Lp(a) levels on revascularization in IHD patients, and the pooled analysis findings are presented in Figure 5. Pooled analysis showed that the hazard of revascularization was significantly higher in patients with high levels of Lp(a) (HR: 1.23, 95% CI: 1.08-1.39, p-value= 0.001). High heterogeneity was reported among the study results (I^2^: 65%). We performed sensitivity analysis to found the source of high heterogeneity despite the fact that all studies showed similar association between Lp(a) and revascularization. By removing the study conducted by Yang et al. [21], the pooled effect remains similar, but the heterogeneity decreased from 65% to 13% (HR: 1.18, 95% CI: 1.10-1.26, p-value: 0.013).

Discussion

This meta-analysis aims to assess the effect of Lp(a) levels of cardiovascular outcomes in patients with IHD. We assessed MACE, myocardial infarction, revascularization, and all-cause mortality. Pooled analysis showed that the hazard of high level of Lp(a) was significantly associated with increased hazard of MACE, myocardial infarction, revascularization, and all-cause mortality. A previous meta-analysis focusing on patients undergoing percutaneous coronary intervention (PCI) found that elevated Lp(a) levels were linked to higher risks of all-cause mortality (risk ratio (RR): 1.26), cardiovascular death (RR: 1.58), myocardial infarction (RR: 1.44), revascularization (RR: 1.38), and stroke (RR: 1.18) [26]. Another meta-analysis demonstrated that each 50 mg/dL increase in Lp(a) raises the risk of coronary heart disease (CHD) by about 30% [27]. Additionally, genetic research has identified that variations at the LPA gene locus correlate with CHD risk, further supporting the causal link between them [28]. The European Atherosclerosis Society guidelines now recommend considering Lp(a) as an emerging risk factor for assessing atherosclerotic cardiovascular disease (ASCVD) risk [29].

Lipoprotein(a) (Lp(a)) is associated with an increased risk of cardiovascular events due to its unique structure and pro-atherogenic, pro-inflammatory, and pro-thrombotic properties [8]. Structurally, Lp(a) consists of an LDL-like particle attached to apolipoprotein(a), which is structurally similar to plasminogen, a key enzyme in the fibrinolytic system [30]. This similarity can interfere with fibrinolysis, leading to reduced breakdown of blood clots and an increased likelihood of thrombus formation. Lp(a) also carries oxidized phospholipids, which promote inflammation within the arterial walls. This inflammatory process contributes to the development of atherosclerotic plaques, which can narrow or block arteries, leading to events such as myocardial infarction or stroke [31]. Additionally, Lp(a) can be taken up by macrophages in the arterial wall, which may result in foam cell formation and further plaque development. Studies have consistently shown that elevated Lp(a) levels correlate with an increased risk of coronary artery disease, ischemic stroke, and peripheral artery disease, making it a significant marker for cardiovascular risk assessment [13-15].

Elevated lipoprotein(a) levels in patients with ischemic heart disease (IHD) have significant clinical implications [32]. As an independent risk factor, high Lp(a) is linked to increased risks of myocardial infarction, stroke, and overall cardiovascular mortality, contributing to residual cardiovascular risk despite optimal management of traditional risk factors. Consequently, assessing Lp(a) levels can aid in identifying IHD patients at higher risk who may benefit from more intensive monitoring or novel therapies targeting Lp(a). With ongoing developments in Lp(a)-lowering therapies, incorporating Lp(a) testing into routine clinical practice could enhance risk stratification and potentially improve long-term outcomes for high-risk IHD patients [33].

Study Limitations

This meta-analysis has some limitations. Firstly, all the studies included were observational, which may introduce selection bias. Secondly, there was limited data available on key variables and outcomes, such as adherence to antiplatelet therapy during follow-up, family history of early atherosclerotic cardiovascular events, and stent thrombosis, which restricted thorough assessment. Additionally, a significant limitation is the potential variability and incomplete understanding of dyslipidemia management across the studies. Dyslipidemia, marked by abnormal blood lipid levels, is a major cardiovascular risk factor, and its effective treatment is vital for reducing adverse outcomes. However, the included studies might have used different therapeutic strategies or lacked detailed documentation on the specific treatments employed for dyslipidemia within their study populations.

## Conclusions

This systematic review and meta-analysis provides compelling evidence that elevated lipoprotein(a) (Lp(a)) levels are significantly associated with adverse cardiovascular outcomes in patients with ischemic heart disease (IHD). The consistently increased risk observed across major adverse cardiovascular events, all-cause mortality, myocardial infarction, and revascularization underscores the potential of Lp(a) as a valuable prognostic marker. These findings highlight the importance of incorporating Lp(a) assessment into routine clinical practice for improved risk stratification in IHD patients. As novel Lp(a)-lowering therapies continue to emerge, this research supports the need for further investigation into targeted interventions for high-risk individuals. Future studies should focus on establishing optimal Lp(a) cutoff values and evaluating the impact of Lp(a) reduction on long-term cardiovascular outcomes, potentially paving the way for more personalized and effective management strategies in IHD.
